# Complete genome sequence and characterization of avian pathogenic *Escherichia coli* field isolate ACN001

**DOI:** 10.1186/s40793-015-0126-6

**Published:** 2016-01-28

**Authors:** Xiangru Wang, Liuya Wei, Bin Wang, Ruixuan Zhang, Canying Liu, Dingren Bi, Huanchun Chen, Chen Tan

**Affiliations:** State Key Laboratory of Agricultural Microbiology, College of Veterinary Medicine, Huazhong Agricultural University, Wuhan, Hubei 430070 China; College of Animal Science & Veterinary Medicine, Huazhong Agricultural University, Wuhan, Hubei 430070 China

**Keywords:** APEC, Colibacillosis, Genome sequencing, Chromosome, Plasmid

## Abstract

Avian pathogenic *Escherichia coli* is an important etiological agent of avian colibacillosis, which manifests as respiratory, hematogenous, meningitic, and enteric infections in poultry. It is also a potential zoonotic threat to human health. The diverse genomes of APEC strains largely hinder disease prevention and control measures. In the current study, pyrosequencing was used to analyze and characterize APEC strain ACN001 (= CCTCC 2015182^T^ = DSMZ 29979^T^), which was isolated from the liver of a diseased chicken in China in 2010. Strain ACN001 belongs to extraintestinal pathogenic *E. coli* phylogenetic group B1, and was highly virulent in chicken and mouse models. Whole genome analysis showed that it consists of six different plasmids along with a circular chromosome of 4,936,576 bp, comprising 4,794 protein-coding genes, 108 RNA genes, and 51 pseudogenes, with an average G + C content of 50.56 %. As well as 237 coding sequences, we identified 39 insertion sequences, 12 predicated genomic islands, 8 prophage-related sequences, and 2 clustered regularly interspaced short palindromic repeats regions on the chromosome, suggesting the possible occurrence of horizontal gene transfer in this strain. In addition, most of the virulence and antibiotic resistance genes were located on the plasmids, which would assist in the distribution of pathogenicity and multidrug resistance elements among *E. coli* populations. Together, the information provided here on APEC isolate ACN001 will assist in future study of APEC strains, and aid in the development of control measures.

## Introduction

The group known as extraintestinal pathogenic *Escherichia coli* (ExPEC), including uropathogenic *E. coli*, neonatal-meningitis *E. coli*, and avian pathogenic *E. coli* (APEC), encompasses *E. coli* strains that cause severe extraintestinal systemic infections such as septicemia, meningitis, and pyelonephritis in both humans and animals. In the veterinary field, APEC mainly causes avian colisepticemia, a widespread infectious disease that leads to significant economic losses in the poultry industry [1, 2]. It has also been widely reported to represent a zoonotic risk, with the potential for spread between animals and humans [3]. However, an incomplete understanding of the genetic features, as well as the genome diversity and frequent occurrence of horizontal gene transfer in APEC, has made it very difficult to carry out pathogenesis studies aimed at preventing APEC infections [4]. Therefore, it is important to explore any useful features within APEC genomes.

Here, we report the full genome sequence and preliminary functional annotation of virulent APEC strain ACN001 (= CCTCC 2015182^T^ = DSMZ 29979^T^), which was isolated from a chicken suffering from avian colibacillosis. The study aimed to characterize the genomic features of strain ACN001 to provide information that will drive further study of APEC to better control its spread.

## Organism information

### Classification and features

**Fig. 1:**
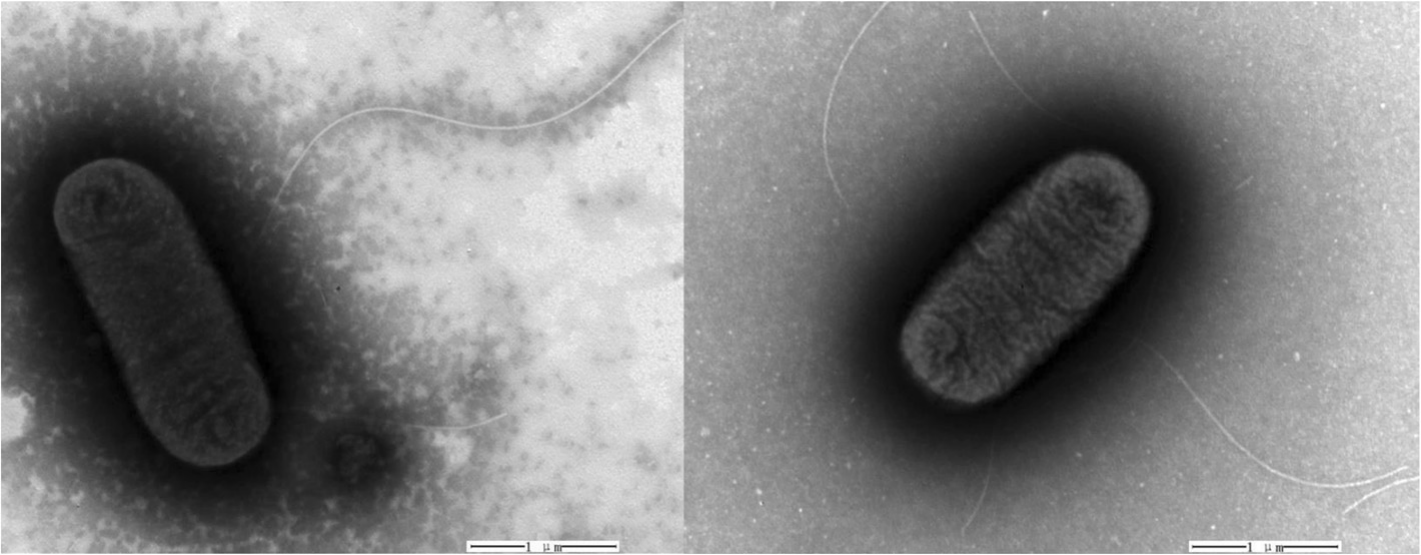
Transmission electron micrograph of APEC strain ACN001 (= CCTCC 2015182^T^ = DSMZ 29979^T^). Strain ACN001 is a short to medium rod-shaped bacterium with a length of 1–2 μm and a diameter of 0.5–0.8 μm. It moves via peritrichous flagella. The magnification rate is 20,000×. The scale bar indicates 1 μm

APEC is a Gram-negative, aerobic and facultatively anaerobic, non-spore forming, short to medium rod-shaped bacterium, which belongs to the *Escherichia* genus of the family *Enterobacteriaceae* (Table 1). It is an etiologic agent of avian colibacillosis, which mainly causes systemic extraintestinal diseases in poultry, including respiratory, hematogenous, meningitic, and enteric infections [5]. Based on previous chicken and mouse models infection studies, APEC strain ACN001 is a highly virulent field isolate, with a length of 1–2 μm and a diameter of 0.5–0.8 μm. It is a mesophile that can grow at temperatures of 10–45 °C, with optimum growth from 37–42 °C (Table 1). It is motile by the means of peritrichous flagella (Fig. 1).

**Table 1:** Classification and general features of APEC strain ACN001

MIGS ID	Property	Term	Evidence code^a^
	Classification	Domain *Bacteria*	TAS [24]
		Phylum *Proteobacteria*	TAS [25]
		Class *Gammaproteobacteria*	TAS [26, 27]
		Order ‘*Enterobacteriales*’	TAS [26, 27]
		Family *Enterobacteriaceae*	TAS [28]
		Genus *Escherichia*	TAS [29, 30]
		Species *Escherichia coli*	TAS [29, 30]
	Gram stain	Negative	TAS [31]
	Cell shape	Rod	TAS [31]
	Motility	Motile	TAS [31]
	Sporulation	None-sporeforming	TAS [31]
	Temperature range	Mesophile	TAS [31]
	Optimum temperature	37 °C	TAS [31]
	pH range; Optimum	5.5–8.0; 7.0	TAS [31]
MIGS-6	Habitat	Host-associated	TAS [1, 32]
MIGS-6.3	Salinity range	Not reported	
MIGS-22	Oxygen requirement	Aerobe and facultative anaerobe	TAS [31, 33]
	Carbon source	Carbohdrates, salicin, sorbitol, mannitol, indole, peptides	TAS [34]
	Energy metabolism	Chemo-organotrophic	TAS [33]
MIGS-15	Biotic relationship	Parasitism	TAS [1, 32]
MIGS-14	Pathogenicity	Pathogenic	TAS [1, 32]
MIGS-4	Geographic location	China	NAS
MIGS-5	Sample collection	2010	NAS
MIGS-4.1	Latitude	Not reported	
MIGS-4.2	Longitude	Not reported	
MIGS-4.3	Depth	Not reported	
MIGS-4.4	Altitude	Not reported	

^a^Evidence codes - TAS: Traceable Author Statement (i.e., a direct report exists in the literature); NAS: Non-traceable Author Statement (i.e., not directly observed for the living, isolated sample, but based on a generally accepted property for the species, or anecdotal evidence); IDA: Inferred from Direct Assay. These evidence codes are from the Gene Ontology project [35]

The 16S rRNA gene sequence of ACN001 was compared with those of other *E. coli* strains available from the GenBank database using BLAST with default settings [6]. The APEC strain ACN001 16S rRNA gene shared an average nucleotide identity of 99.61 % with the corresponding regions of published strains, including *E. coli* UMNK88 (GenBank accession no. CP002729.1, 100 %), *E. coli* 12009 (AP010958.1, 100 %), *E. coli* BL21(DE3) (CP001509.3, 100 %), *E. coli*ATCC 8739 (CP000946.1, 100 %), *E. coli* APEC O78 (CP004009.1, 100 %), *E. coli* IAI39 (CU928164.2, 99 %), *E. coli* UMN026 (CU928163.2, 99 %), *E. coli* K12substr. MG1655 (U00096.3, 99 %), *E. coli* K12substr. DH10B (CP000948.1, 99 %), *E. coli* K12substr. W3110 (AP009048.1, 99 %), *E. coli* APEC O1 (CP000468.1, 99 %), *E. coli* CFT073 (AE014075.1, 99 %), and *E. coli* UTI89 (CP000243.1, 99 %).

**Fig. 2:**
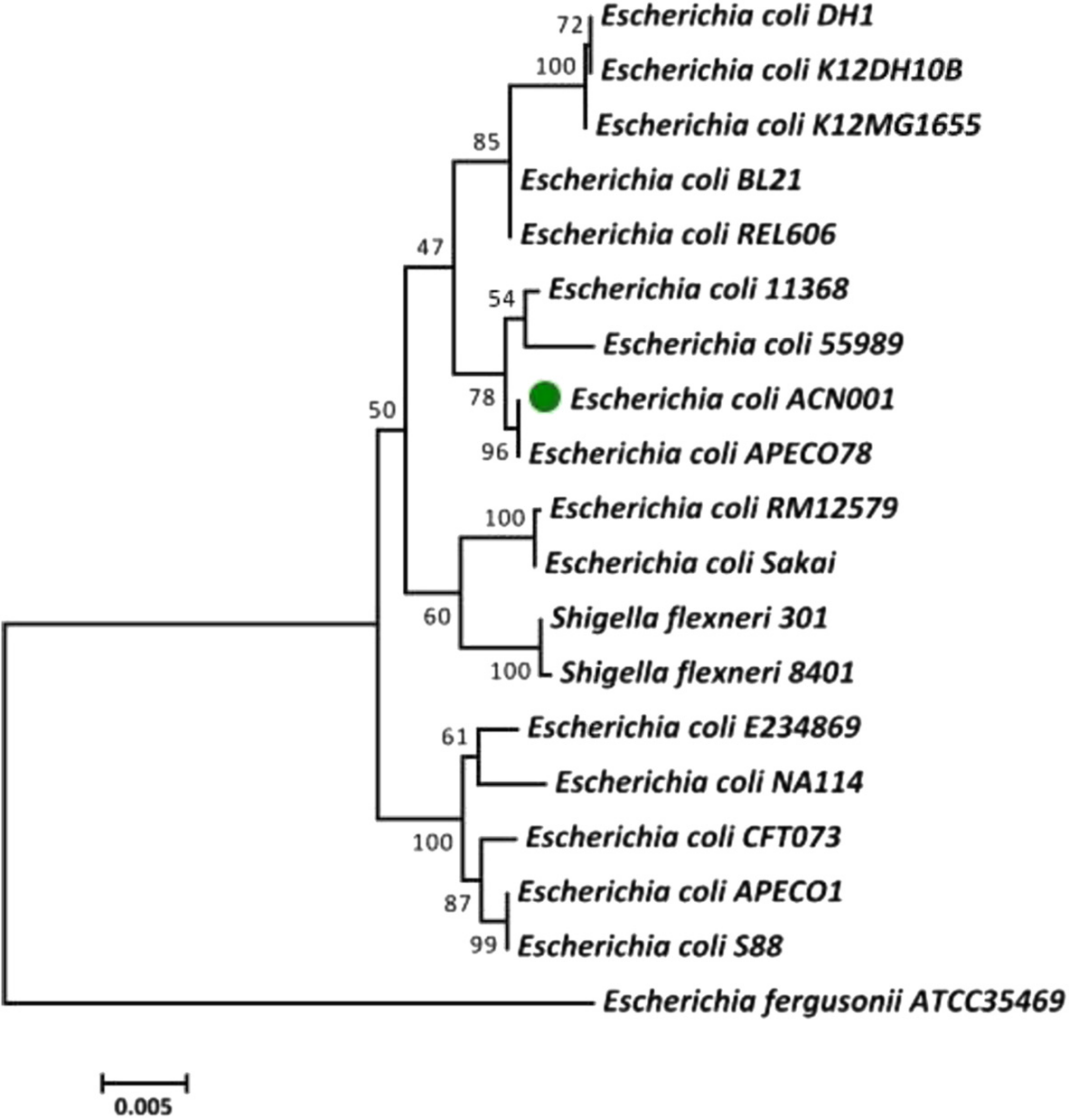
Phylogenetic tree highlighting the position of ACN001 relative to other strains within the *Enterobacteriaceae* family. The phylogenetic tree was constructed based on seven housekeeping genes (*adk, fumC, gyrB, icd, mdh, purA, and recA*) according to the aligned gene sequences using maximum likelihoods derived from MEGA software version 5. The scale bar represents the divergence time of different strains. *Escherichia fergusonii* (ATCC35469) was used as an out-group

ExPEC strains can be divided into different *E. coli* Collection Reference phylogroups (A, B1, B2, D and E) according to the sequences of housekeeping genes (e.g., *adk, fumC, gyrB, icd, mdh, purA, and recA*) [7, 8]. We constructed a phylogenetic tree based on the aligned gene sequences using a maximum likelihood approach and MEGA (version 5), with 1,000 randomly selected bootstrap replicates [7] (Fig. 2). ACN001 belonged to phylogenetic group B1, and was located on the same branch as APEC O78, another highly virulent group B1 strain [7]. Genes from the following strains were used to construct the phylogenetic tree: *E. coli* DH1 (GenBank accession no. NC_017625), *E. coli* K12 DH10B (NC_010473), *E. coli* K12 MG1655 (NC_000913), *E. coli* BL21 (NC_012947), *E. coli* REL606 (NC_012967), *E. coli* 11368 (NC_013361), *E. coli* 55989 (NC_011748), *E. coli* APEC O78 (NC_020163), *E. coli* RM12579 (NC_017656), *E. coli* Sakai (NC_002695), *Shigella flexneri* 301 (NC_004337), *Shigella flexneri* 8401 (NC_008258), *E. coli* E234869 (NC_011601), *E. coli* NA114 (NC_017644), *E. coli* CFT073 (NC_004431), *E. coli* APEC O1 (NC_008563), *E. coli* S88 (NC_011742). *Escherichia fergusonii*ATCC35469 (NC_011740) was used as an out-group.

## Genome sequencing and annotation

### Genome project history

APEC strain ACN001 was selected for whole genome sequencing at the Chinese National Human Genome Center in Shanghai, China, because of its high virulence and potential zoonotic risk. Sequence assembly and annotation were completed in December 2012, and the complete genome sequence was deposited in GenBank under accession number CP007442. A summary of the project information and its association with “Minimum Information about a Genome Sequence” [9] are provided in Table 2.

**Table 2:** Genome sequencing project information

MIGS ID	Property	Term
MIGS-31	Finishing quality	Finished
MIGS-28	Libraries used	454 Titanium paired-end library (800-bp insert size)
MIGS-29	Sequencing platforms	454-GS-FLX-Titanium
MIGS-31.2	Fold coverage	23.58×
MIGS-30	Assemblers	Newbler version 2.3
MIGS-32	Gene calling method	GeneMark, Glimmer
	Locus tag	J444
	GenBank ID	CP007442
	GenBank Date of Release	09-July-2015
	BIOPROJECT	PRJNA234088
MIGS-13	Source Material Identifier	CCTCC 2015182
	Project relevance	Pathogenic bacterium, biotechnological

### Growth conditions and genomic DNA preparation

APEC strain ACN001 was cultivated on LB medium as previously described [1]. High quality genomic DNA for sequencing was extracted using a cetyl trimethyl ammonium bromide (CTAB) method, and the concentration and purity were determined by agarose gel electrophoresis.

### Genome sequencing and assembly

The complete genome of APEC strain ACN001 was sequenced using the Roche 454 GS-FLX platform (Roche, Basel, Switzerland). Each library fragment had an average size of 800 bp, yielding 215,687 reads corresponding to approximately 166.3 Mb, and providing 23.58-fold coverage of the genome. The reads were assembled into 245 contigs using Newbler version 2.3 (454 Life Sciences, Branford, CT), including 195 large contigs that were > 500 bp. Sequence data from APEC strains O1 and O78, which showed a high level of similarity to the ACN001 draft sequences, was used as a reference for the finishing process. All large contigs were analyzed using Cytoscape software version 2.8.2 to determine their relative positions. Gap closure between large contigs was completed by sequencing potential neighboring contigs using an ABI 3730xl sequencer (Applied Biosystems). The Phred/Phrap/Consed software package was used for sequence assembly and quality assessment in the subsequent finishing process. Possible misassemblies were corrected by sub-cloning and sequencing bridging PCR fragments. A total of 217 additional amplification reactions were performed to close all gaps and improve the quality of the sequences. The error rate of the final ACN001 genome sequence was less than 10^−5^.

### Genome annotation

The complete ACN001 genome sequence was analysed using Glimmer 3.0 [10, 11] and GeneMark [12, 13] for gene prediction, the tRNAscan-SE tool for tRNA identification [14], and RNAmmer [15] for ribosomal RNA identification. The predicted protein-coding genes were translated into amino acid sequences and annotated using the NCBI and UniProt non-redundant sequence databases [16], the Kyoto Encyclopedia of Genes and Genomes database [17], and, subsequently, the Cluster of Orthologous Genes database [18] to identify the specific protein products and their functional categories. Additional gene analysis and miscellaneous features were predicted using TMHMM [19], SignalP [20], and the Rapid Annotation using Subsystem Technology server databases [21]. Clustered regularly interspaced short palindromic repeat elements were detected using CRT [22] and PILER-CR [23].

## Genome properties

**Fig. 3:**
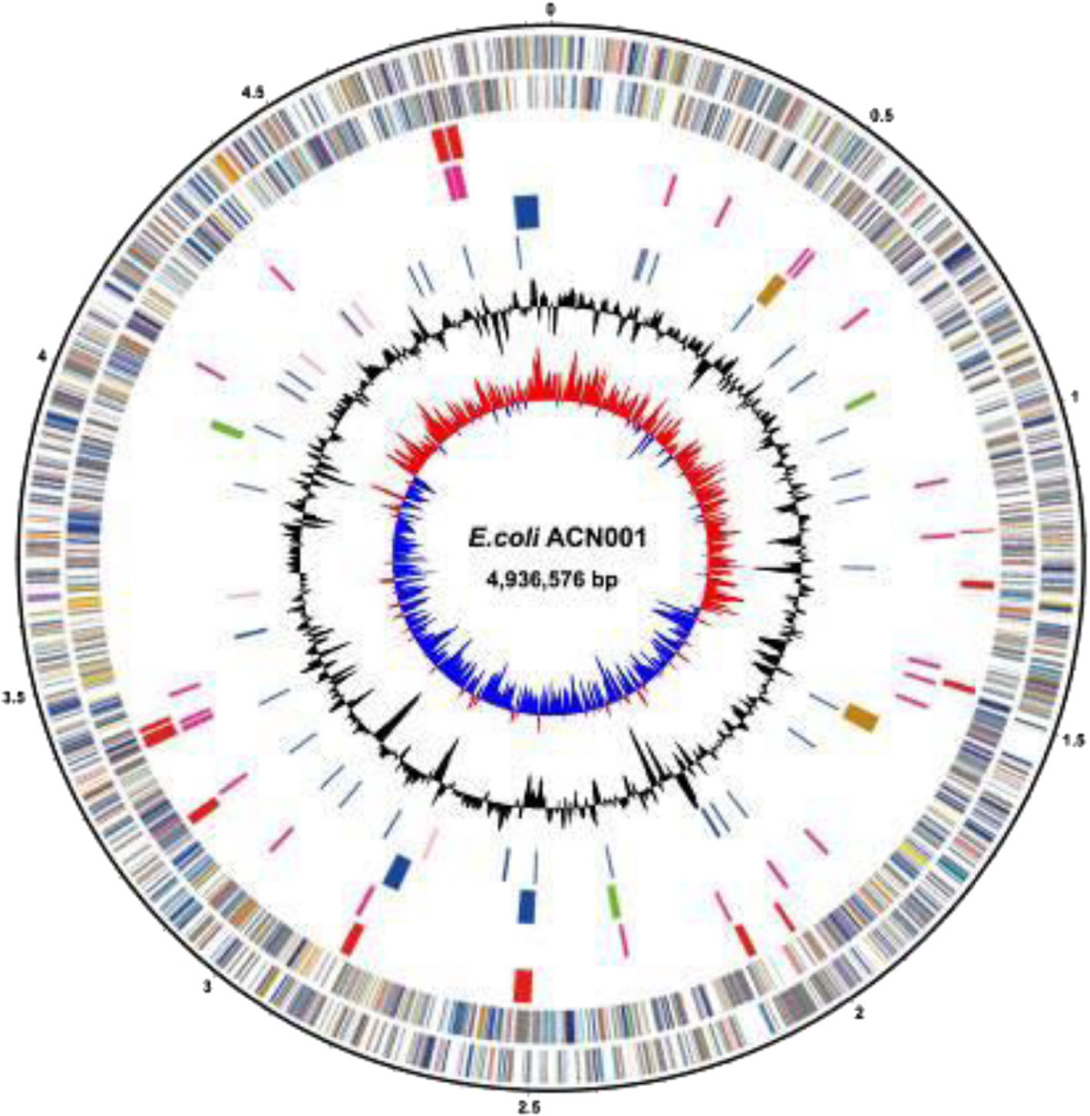
Graphical map of the APEC strain ACN001 chromosome. Circular representation of the ACN001 chromosome displaying relevant genomic features. From outside to the center: genes on the forward strand (colored by COG category), genes on the reverse strand (colored by COG category), insertion sequences (ISs), genomic islands (GIs), prophage sequences, RNAs, GC content and GC skew

**Fig. 4:**
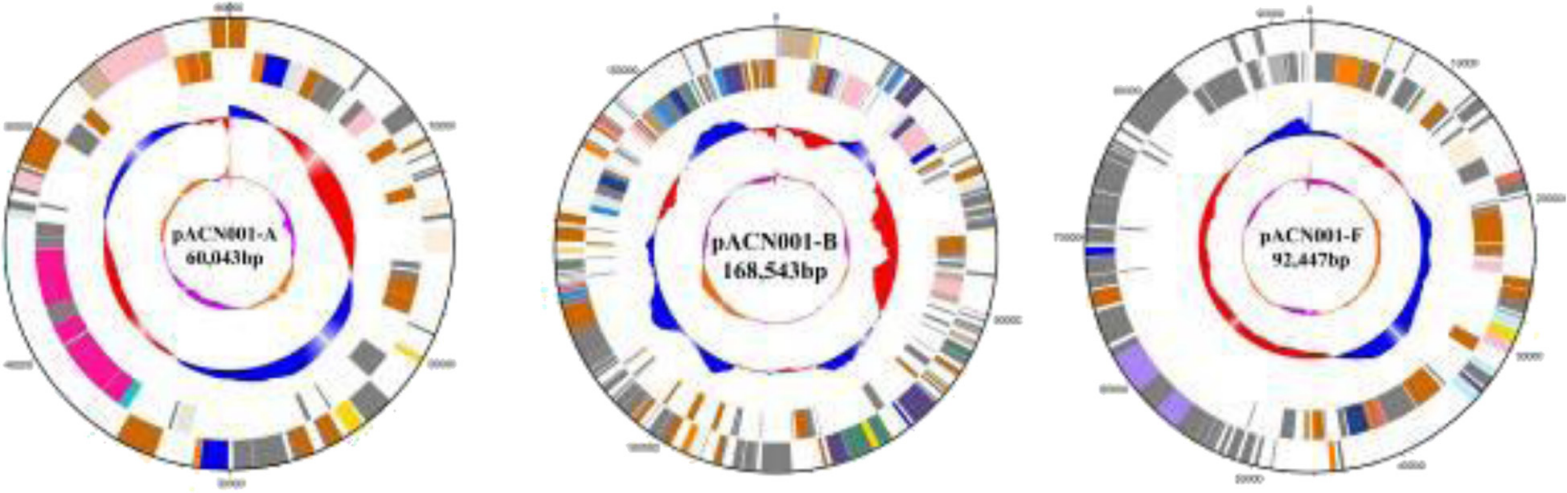
Graphical map of the large plasmids in APEC strain ACN001. Circular representation of ACN001 plasmids pACN001-A, B, and F displaying relevant features. From outside to the center: genes on the forward strand (colored by COG category), genes on the reverse strand (colored by COG category), GC content, GC skew. Order and size from left to right: pACN001-A, 60,043 bp; pACN001-B, 168,543 bp; pACN001-F, 92,447 bp

The complete genome of ACN001 comprises one circular chromosome of 4.9 Mb in size (4,936,576 bp, Fig. 3) and six plasmids: pACN001-A (60,043 bp), pACN001-B (168,543 bp), pACN001-C (5,784 bp), pACN001-D (6,747 bp), pACN001-E (6,822 bp) and pACN001-F (92,447 bp) (Fig. 4 and Table 3), with an average G + C content of 50.56 % (Table 4). A total of 5,253 genes were predicted in the genome, of which 4,794 coded for proteins, 108 were RNA-related, and 51 were pseudogenes. A total of 3,630 (69.11 %) of the protein-coding genes were assigned specific functions, with hypothetical functions assigned to the remaining genes. The genome properties are presented in Tables 3 and 4, and Figs. 3 and 4. The COG functional categories are listed in Table 5.

**Table 3:** Summary of ACN001 genome: one chromosome and six plasmids

Label	Size (Mb)	Topology	INSDC identifier
Chromosome	4.9	Circular	GenBank CP007442
Plasmid pACN001-A	0.06	Circular	GenBank KC853434
Plasmid pACN001-B	0.17	Circular	GenBank KC853435
Plasmid pACN001-C	0.006	Circular	GenBank KC853436
Plasmid pACN001-D	0.007	Circular	GenBank KC853437
Plasmid pACN001-E	0.007	Circular	GenBank KC853438
Plasmid pACN001-F	0.09	Circular	GenBank KC853439

*INSDC* International Nucleotide Sequence Database Collaboration

**Table 4:** Genome statistics

Attribute	Value	% of Total^a^
Genome size (bp)	5,276,962	100.00
DNA coding (bp)	4,614,690	87.45
DNA G + C (bp)	2,669,115	50.56
Total genes	5,253	100.00
Protein coding genes	4,794	91.26
RNA genes	108	2.06
tRNA genes	86	1.64
rRNA genes	22	0.42
Pseudo genes	51	0.97
Genes with function prediction	3,630	69.11
Genes assigned to COGs	3,203	60.97
Genes with Pfam domains	4,463	84.96
Genes with signal peptides	424	8.07
Genes with transmembrane helices	1,124	21.40
CRISPR repeats	2	

^a^Total based on either the size of the genome in base pairs (bp) or the total number of genes in the annotated genome

**Table 5:** Number of genes associated with general COG functional categories

Code	Value	% age	Description
J	177	3.37	Translation, ribosomal structure and biogenesis
A	2	0.04	RNA processing and modification
K	205	3.90	Transcription
L	197	3.75	Replication, recombination and repair
B	0	0.00	Chromatin structure and dynamics
D	41	0.78	Cell cycle control, Cell division, chromosome partitioning
Y	0	0.00	Nuclear structure
V	56	1.07	Defense mechanisms
T	90	1.71	Signal transduction mechanisms
M	187	3.56	Cell wall/membrane biogenesis
N	106	2.02	Cell motility
Z	0	0.00	Cytoskeleton
W	0	0.00	Extracellular structures
U	48	0.91	Intracellular trafficking and secretion
O	116	2.21	Posttranslational modification, protein turnover, chaperones
C	256	4.87	Energy production and conversion
G	286	5.44	Carbohydrate transport and metabolism
E	318	6.05	Amino acid transport and metabolism
F	84	1.60	Nucleotide transport and metabolism
H	128	2.44	Coenzyme transport and metabolism
I	76	1.45	Lipid transport and metabolism
P	188	3.58	Inorganic ion transport and metabolism
Q	62	1.18	Secondary metabolites biosynthesis, transport and catabolism
R	309	5.88	General function prediction only
S	332	6.32	Function unknown
-	1,993	37.94	Not in COGs

## Insights from the genome sequence

APEC infection causes significant economic losses to the poultry industry. An incomplete understanding of the APEC genome complexity impedes the study of pathogenesis and subsequent development of control measures. Here, complete genome sequencing and annotation of APEC virulent isolate ACN001 was carried out, which identified 4,794 protein-coding genes, accounting for 91.26 % of the total number of genes (5,253 genes). Notably, preliminary sequence analysis revealed 39 insertion sequences (ISs), 12 predicated genomic islands (GIs), 8 prophage-related sequences and 2 CRISPR elements. These elements involved 237 coding sequences on the circular chromosome, indicating possible genetic crosstalk among *E. coli* populations. These elements might represent the genetic differences between ACN001 and other APEC strains, and reflect the potential interactions of this strain with the environment. Further comparative approaches will be applied to help to better elucidate the interrelationship of these traits with certain phenotypes, such as adaptability and pathogenicity.

Moreover, six plasmids were found in strain ACN001, including three large plasmids (pACN001-A, B, and F) and three small plasmids (pACN001-C, D, and E). Antibiotic resistance genes and the majority of the essential virulence genes were located on the three large plasmids, while only 8, 9 and 10 protein-coding genes with unknown functional annotations were found on plasmids pACN001-C, D, and E, respectively. The location of antibiotic resistance and virulence genes on plasmids in strain ACN001 may allow the propagation of multidrug resistance and virulence factors among *E. coli* populations in poultry.

## Conclusion

This study presents the whole genome sequence of APEC strain ACN001, a chicken-derived isolate causing typical avian colibacillosis. The genome of ACN001 consists of a circular chromosome containing 4,794 protein-coding genes and 108 RNA genes, along with six plasmids with different features. We observed 39 ISs, 12 predicated GIs, 8 prophage-related sequences and 2 CRISPR elements on the chromosome, suggesting frequent genetic crosstalk, such as horizontal gene transfer, between ACN001 and other *E. coli* populations. Among the six plasmids identified in this strain, three large plasmids contained multiple antibiotic resistance and virulence genes, while the three small plasmids contained genes with unknown functional annotations. These plasmid-borne pathogenicity-associated features should be closely monitored to prevent further spread amongst the diverse *E. coli* populations, especially APEC. The genome sequencing and annotation of virulent APEC isolate ACN001 provides valuable genetic information for future study of the pathogenesis of APEC strains, which will help in the development of prevention and control measures.

## Abbreviations

APEC: Avian pathogenic *Escherichia coli*
ExPEC: Extraintestinal pathogenic *Escherichia coli*
